# Performance of Clinical and Biochemical Parameters in Identifying Renal Histopathology and Predictors of One-Year Renal Outcome in Lupus Nephritis—A Single Centre Study from India

**DOI:** 10.3390/diagnostics12123163

**Published:** 2022-12-14

**Authors:** Aishwarya Gopal, Chengappa Kavadichanda, Devender Bairwa, Sanket Shah, Sonal Mehra, Bheemanathi Hanuman Srinivas, Christina Mary Mariaselvam, Molly Mary Thabah, Vir Singh Negi

**Affiliations:** 1 Department of Clinical Immunology, Jawaharlal Institute of Postgraduate Medical Education and Research, Puducherry 605006, India; 2 Department of General Medicine, All India Institute of Medical Sciences, Bilaspur 174001, India; 3 GCS Medical College, Ahmedabad 380025, India; 4 Centre for Spine and Rheumatology, Delhi 110058, India; 5 Department of Pathology, Jawaharlal Institute of Postgraduate Medical Education and Research, Puducherry 605006, India; 6 All India Institute of Medical Sciences, Bilaspur 174001, India

**Keywords:** chronic kidney disease, SLE, Lupus Nephritis, proteinuria, histopathology, prognosis

## Abstract

**Objectives:** To assess the performance of clinical and biochemical parameters in identifying renal histopathology. To assess the performance of a combination of demographic, clinical, serological and histopathological parameters in determining renal response at one year. **Methods:** Data of biopsy-proven (ISN/RPS—2003 criteria) Lupus Nephritis (LN) were extracted from the institute database. Demographic, clinical and biochemical parameters at the time of biopsy were noted, and their associations with histopathological class, activity and chronicity scores were evaluated. Follow-up data at one year were collected. Complete, partial or no response (CR, PR, NR) for renal outcomes at one year and the predictors of NR were assessed. **Results:** Out of the 333 renal biopsies, 240 (71.8%) were Class III/IV. More patients with Class III/IV LN had hypertension (52.1%) and low eGFR (*p* < 0.001). Among Class III/IV, AS correlated weakly with UPCR (r = 0.31, *p* < 0.01), eGFR (r = −0.172; *p* < 0.01) and CS with eGFR (r = −0.212; *p* < 0.01). The presence of either hypertension, UPCR > 0.5 g/day, active urinary sediments or serum creatinine >1.3 g/dL had a sensitivity of >96% and specificity of <9% in detecting proliferative LN, crescents, interstitial inflammation and chronicity. NR was higher in males (aOR:3.9, 95% CI:1.4–11.0, *p* < 0.001), those with abnormal baseline creatinine (aOR: 1.9, 95% CI: 1.1–3.2, *p* < 0.001), higher renal SLEDAI (*p* < 0.05), higher AS, CS (*p* < 0.001) and interstitial inflammation (*p* < 0.005). In the binary logistic regression, the combination of male sex, baseline creatinine, UPCR and CS performed best in predicting NR (AUC: 0.762; 95% CI: 0.684–0.840, *p* < 0.001). **Conclusions:** Clinical and biochemical parameters alone have a poor specificity in identifying renal histopathology. A combination of demographic, clinical and histopathology parameters can better predict renal outcomes at one year.

## 1. Introduction

Lupus nephritis (LN) is one of the most important manifestations of Systemic Lupus Erythematosus (SLE), contributing to significant morbidity and mortality [1]. The diagnosis of LN is routinely based on a combination of clinical features, serum and urine biochemistry, and histopathological analysis of the kidney. Although various guidelines agree that renal biopsy is essential and serves as the cornerstone for the diagnosis and treatment decision, there are often arguments for and against performing renal biopsy at the practice level. The main barriers to conducting renal biopsies are operator expertise, availability of ultrasound facility, exposure to biopsy during training, concerns about biopsy-related complications and patient preferences [2,3]. Attempts to substitute renal biopsy with non-invasive serum indicators for LN diagnosis have been unsuccessful [4].

Proliferative LN, which includes Classes III and IV, mandates intensive immunosuppression with either cyclophosphamide or mycophenolate-based regimens [5,6,7]. However, despite intensive immunosuppressive therapy, only <60% of individuals achieve remission, and around 30% of LN cases progress to chronic kidney disease [6,7,8,9]. The strongest factor predicting good long-term renal outcomes is the reduction in proteinuria at 12 months [10,11]. Thus, achieving the proteinuria target by 12 months seems to be a proxy for long-term outcomes. This concept again questions the role and requirement for histopathological analysis in LN in determining the long-term outcome.

Previous retrospective studies have shown that active and chronic lesions, including those in the tubulointerstitial compartment in renal histopathology, were significant determinants of long-term renal outcomes [10]. These studies, however, were not comprehensive, given their limited sample size. Analysis of the histopathology or the clinical characteristics in isolation is apparently inadequate in determining the long-term outcome.

Likewise, there is a lack of robust studies assessing the performance of the routinely used clinical, biochemical findings and their association with various histological findings in the kidney. Thus, there is a need to systematically analyse the performance of the routinely available clinical and biochemical parameters in accurately identifying the histopathological changes and their role in determining long-term outcomes in LN. Previous retrospective studies reported were predominantly in Caucasians [12,13]. Hence, it is crucial to study this in other ethnic groups, such as Africans and Asians, among whom LN is considered to be more severe [14,15].

Therefore, the objectives of this study were to assess whether the clinical and biochemical parameters at baseline rightly identified renal histopathological findings, as well as to assess the performance of a combination of demographic, clinical, serological and histopathological parameters in determining renal response at one year in an ethnically homogenous population.

## 2. Materials and Methods

This is a retrospective cohort study conducted in a tertiary care teaching institute in India. All patients with biopsy-proven LN between 2012 and 2020 at the Jawaharlal Institute of Postgraduate Medical Education and Research and who received treatment with either Cyclophosphamide (CYC)- or Mycophenolate Mofetil (MMF)-based induction regimen for Class III/IV/V LN were retrospectively included in the study. Renal histopathology findings were classified according to the International Society of Nephrologists (ISN)/Renal Pathologic Society (RPS) classification 2003 reported by a qualified nephropathologist [16]. The activity and chronicity scores according to a modified NIH index [17] were adapted from the biopsy reports. Those with Class III or Class IV LN were grouped as proliferative LN, and those with Class III/IV + Class V were grouped separately as a combined class. The presence of crescents, interstitial inflammation, fibrinoid necrosis and any vascular changes were reported by the pathologist and noted separately for analysis. Missing data were excluded from the analyses.

Demographic variables, including age at onset of SLE, age at onset of LN, gender, total duration of SLE and duration of follow-up, were collected. Baseline clinical and laboratory parameters, including hypertension (defined as either systolic blood pressure > 140 mmHg or diastolic blood pressure >90 mmHg, or both), serum creatinine, estimated glomerular filtration rate (eGFR) according to Chronic Kidney Disease Epidemiology Collaboration (CKD-EPI), serum albumin, urine protein creatinine ratio (UPCR), active urinary sediments [18], defined as red blood cells (RBCs) > 5cells/high power field (HPF) or white blood cells (WBC) > 5cells/HPF, or cellular casts that were attributed to SLE, were noted. Disease activity was assessed using the SLE disease activity index-2K (SLEDAI -2K) [19] and Renal SLEDAI. The serological parameters collected included complement C3 and C4 levels quantified by nephelometry, antibodies to double stranded deoxyribonucleic acid (anti-dsDNA) and antiphospholipid antibodies (aPLs: anticardiolipin antibody (ACLA) IgG/IgM; anti-beta-2 glycoprotein antibody (B2GPI) IgG/IgM; Lupus Anticoagulant (LAC)) quantified by the Enzyme-linked immune sorbent assay.

Patients with three-monthly follow-up data for a minimum of twelve months were included in the study. Renal response was defined as complete response (CR), partial response (PR) and no response (NR) according to the EULAR/EDTA recommendations 2020 [8]. All patients with proliferative LN had received either cyclophosphamide- or MMF-based immunosuppression. Those who needed rescue therapy or a change of immunosuppression due to lack of response were also classified as NR. Relapse was defined as the reappearance of any of the following: proteinuria with UPCR > 0.5, presence of active urinary sediments or new onset renal dysfunction attributed to SLE. The number of deaths and losses to follow-up were noted. Those who did not have follow-up records for more than six months were considered lost to follow-up.

This study was approved by the Institutional Ethics Committee (JIP/IEC/2018/003). The study was conducted as per the Indian Council for Medical Research (ICMR) Guidelines for Biomedical Research on Human Participants and in accordance with the ethical standards laid down in the 1964 Declaration of Helsinki and its later amendments.

### Statistical Analysis

The categorical variables were represented as frequency and percentages. All continuous variables were tested for normality using the Kolmogorov–Smirnov test. Normally distributed variables were summarised as the mean ± standard deviation (SD) and non-normally distributed variables as median with inter-quartile range (IQR). The associations of categorical variables between the different histopathological classes were tested using the Chi-square test, and those of continuous variables were tested using the Kruskal–Wallis ANOVA for three groups, followed by pairwise comparisons and Mann–Whitney U-test for two groups. The sensitivity, specificity, positive and negative predictive values of all the baseline parameters, alone and in combination, in detecting proliferative LN, activity and chronicity were calculated. Correlation analysis was performed, and Spearman’s correlation coefficient was noted to assess the linear relationship. Univariate and multivariate logistic regression to predict NR at one year was performed using the variables that had *p* < 0.1 in the univariate analysis. A receiver operating curve (ROC) was plotted to find out the discriminative ability of the predictive model with pertinent variables. Sensitivity analysis was performed by including the patients lost to follow-up, and the performance of the regression model was re-tested. All analyses were performed using SPSS, version 19 (SPSS for Windows, Version 19.0. Chicago, IL, USA, SPSS Inc.). The correlation graphs were generated using StataSE 13. Sensitivity and specificity calculations were performed using MedCalc software Version 20.110. The analyses were performed at a 5% significance level, and a *p*-value < 0.05 was considered statistically significant.

## 3. Results

### 3.1. Patient Characteristics

**Figure 1:**
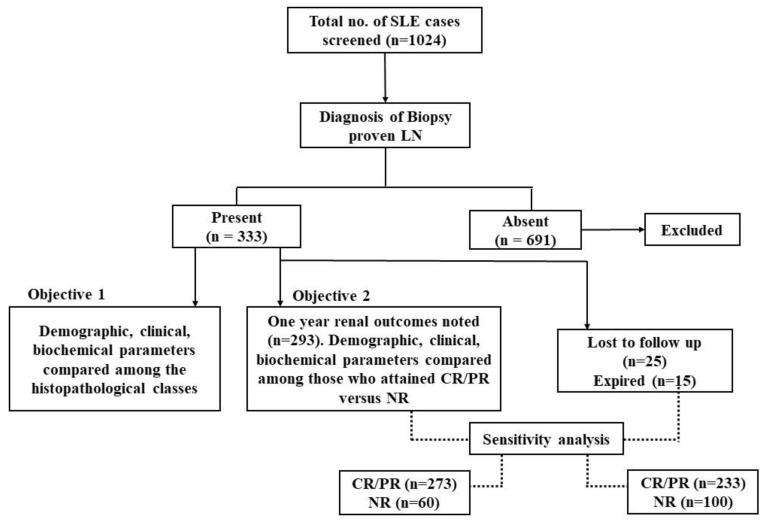
Flow chart showing the disposition of study participants.

Out of 1024 SLE patients screened, a total of 333 patients who underwent renal biopsy at the onset of LN were included in the study (Figure 1). The mean age ± SD at the onset of nephritis was 26.5 ± 12 years, and 92% were females. Class III/IV LN (74%) was the most common, followed by Class I/II (15%), Class V (7.5%) and combined (III/IV + V) Classes (3.5%). 

### 3.2. Correlation and Performance of Clinical Parameters with the Renal Histopathology at Baseline

The baseline characteristics of patients with different biopsy-based LN classes are detailed in Table 1. When compared to the other classes, Class III/IV LN patients had a significantly higher prevalence of hypertension (54%; *p* = 0.001), active urinary sediments (64.2%; *p* = 0.006) and lower eGFR (median, IQR = 87.6, (62.7–118.8), *p* < 0.001). Nephrotic range proteinuria was seen in 32% of Class V and 21% of Class III/IV patients (*p* = 0.004). The serum albumin and serological parameters, such as C3, C4, anti-dsDNA and any aPL positivity, were not different between the different biopsy classes. Class III/IV LN had a significantly higher renal SLEDAI (median, IQR = 12(8–12), *p* < 0.001) compared to Class II and Class V.

### 3.3. Performance of the Clinical and Biochemical Parameters in Identifying the Renal Histopathology

The sensitivity and specificity of hypertension, baseline UPCR > 0.5, active urinary sediments and renal dysfunction (serum creatinine > 1.3 mg/dL), alone and in combination, for differentiating between proliferative and non-proliferative LN and identifying interstitial inflammation, cellular crescents and chronicity, were calculated (Table 2 and Supplementary Table S1). The presence of either hypertension, UPCR > 0.5, active sediments or serum creatinine > 1.3 had a higher sensitivity and poor specificity in detecting Class II/IV LN (97.0% and 6.4%), cellular crescents (98.4% and 4.5%), interstitial inflammation (94.5% and 3.16%) and chronicity (95.8 and 3.85%).

**Figure 2:**
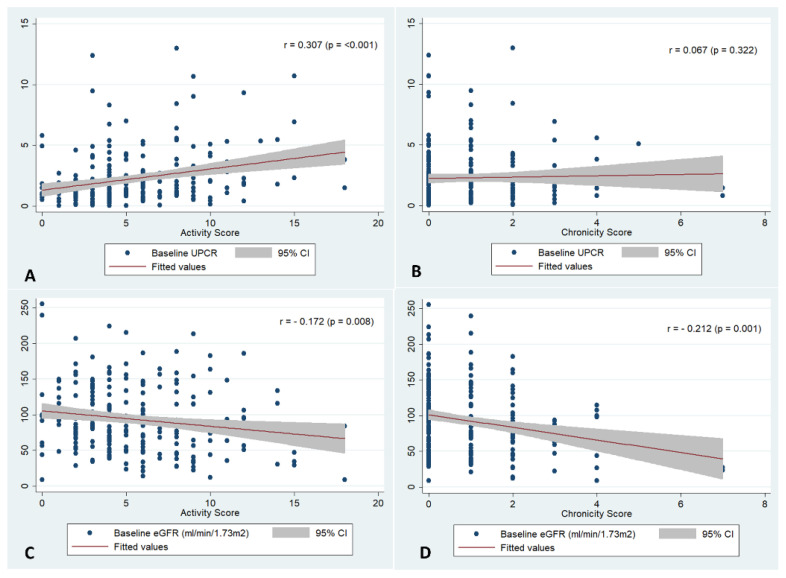
Correlation between (**A**) activity score and baseline UPCR; (**B**) Chronicity score and Baseline UPCR; (**C**) Activity score and Baseline eGFR; (**D**) chronicity and baseline eGFR; eGFR estimated glomerular filtration rate; UPCR Urine protein creatinine ratio.

Among Class III/IV, the activity score had a weak positive correlation with baseline UPCR (r = 0.307, *p* < 0.001)) and negative correlation with eGFR (r = −0.172, *p* < 0.001), while the chronicity score had a weak negative correlation with eGFR (r = −0.212, *p* < 0.001) (Figure 2). Baseline median UPCR was significantly higher among those who had crescents, fibrinoid necrosis and interstitial inflammation (*p* < 0.05, Supplementary Table S2), but the other parameters were similar.

### 3.4. Renal Outcomes at One Year

At the end of one year of treatment, renal outcome was assessed for 293 patients, out of whom 71%, 9% and 20% achieved CR, PR and NR, respectively. In the univariate analysis, NR was higher in males (OR:4.6, 95% CI:1.9–10.8, *p* = 0.001), and the biochemical parameters that were significantly associated with NR were abnormal serum creatinine, the presence of active urinary sediments and higher renal SLEDAI. The titres of anti-dsDNA, C3/C4 levels and aPL positivity were not different between responders and non-responders. The baseline biopsy findings significantly associated with NR at one year were higher activity score (*p* = 0.001), higher chronicity score (*p* = 0.001), interstitial inflammation (*p* = 0.004) and tubular atrophy (*p* = 0.003) (Table 3). Baseline activity and chronicity scores had a weak positive correlation with UPCR at one year (Supplementary Figure S1).

### 3.5. Model to Predict Non-Response at One Year

**Figure 3:**
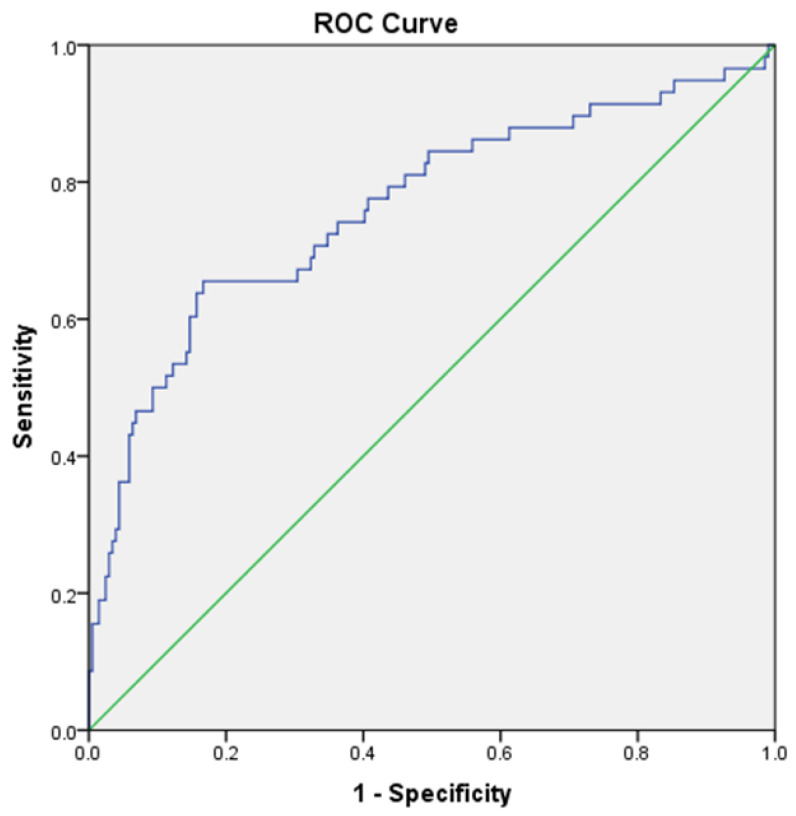
The receiver operator characteristics (ROC) curve and the area under the curve (AUC) for model—Parameters: Baseline serum creatinine, urine PCR, male sex, chronicity score; AUC = 0.762, 95% CI (0.684–0.840), *p* < 0.001.

Univariate and multivariate binary logistic regression analyses were performed to identify the predictors of NR (Table 3 and Table 4). The ROC curve depicts the performance of the model after multivariate logistic regression. The combined clinical–histopathological model comprising baseline serum creatinine, UPCR, male sex and chronicity score performed best in predicting NR (AUC: 0.762, 95% CI: 0.684–0.840 *p* < 0.001) (Figure 3).

### 3.6. Sensitivity Analysis

A sensitivity analysis was performed to account for the missing variables (lost to follow-up (n = 25) and deaths (n = 15)). First, all the patients (n = 35) were considered non-responders. With this assumption, the combined clinico-histopathological model comprising baseline serum creatinine, UPCR, male sex and chronicity score had an AUC of 0.757, 95% CI: 0.680–0.835, *p* < 0.001. Second, all the patients (n = 35) were considered responders. This assumption resulted in a combined clinico-histopathological model with an AUC of 0.692, 95% CI:0.618–0.735, *p* < 0.001.

## 4. Discussion

In this study, we first evaluated whether the baseline clinical and biochemical parameters could predict the renal histopathological changes at the onset of LN. We then analysed the factors predicting renal response at one year. We found that baseline clinical and biochemical parameters alone have a poor specificity in identifying the renal histopathological class, and a combined clinico-histopathological model, which comprised male sex, baseline creatinine, UPCR and the chronicity score, fairly predicted non-response in LN at one year.

We found that parameters such as hypertension, higher serum creatinine and UPCR and the presence of active urinary sediments were more common in proliferative LN. According to the latest EULAR/EDTA recommendations for LN, renal biopsy should be considered when there is evidence of kidney involvement in the form of persistent proteinuria ≥0.5 g/24 h (or UPCR ≥500 mg/g) or an unexplained decrease in eGFR [8]. However, in the presence of only minor renal abnormalities (proteinuria <1 g/day without renal dysfunction, hypertension or active urinary sediments), clinicians prefer not to perform kidney biopsy. In addition, with the use of MMF for induction of all severe classes of LN (Class III/IV or Class V) [5], the need to differentiate between the renal biopsy classes has become less important. Nevertheless, it is important to note that even in the absence of clinical or biochemical signs of renal involvement [20], proliferative LN has been observed in patients with SLE.

In this study, except for renal dysfunction (defined as serum creatinine > 1.3 mg/dL), the specificity of each of the parameters, viz., hypertension, proteinuria (UPCR ≥ 0.5) and the presence of active urinary sediments, in identifying proliferative versus non-proliferative LN was very low. We found that sensitivity was very high when any one parameter was present, but specificity was very low. However, specificity was high when all the above four parameters were present, but in clinical practice, it is rare to find patients having all four parameters (9%). The current recommendations state that less severe forms of LN, such as Class I/II or Class V, without renal dysfunction or a nephrotic range of proteinuria do not need immunosuppressive therapy in the absence of severe extrarenal manifestations that warrant the same [8]. Due to the poor specificity of clinical and biochemical parameters in identifying the histopathological class, there is a possibility of administering inappropriate immunosuppressive therapy to patients who might have less severe forms of LN if a renal biopsy is not performed at the onset. Moroni et al. showed that a delay between the onset of clinical manifestations of renal disease and renal biopsy is an independent risk factor for poor renal outcomes [12]. 

We observed that, among Class III/IV LN, the baseline UPCR and activity scores showed a weak positive correlation, while eGFR and activity scores, as well as eGFR and chronicity scores, showed a weak negative correlation. In a study by Nasri et al. [21], a positive correlation between serum creatinine and percentage of activity, chronicity and proportion of glomeruli with crescents was reported. They also observed a significant relationship between chronicity and proteinuria; however, this association with activity was insignificant. The proportion of proliferative LN with crescents in our study was similar to that reported by Yu et al. (27% and 22%, respectively). Higher serum creatinine was significantly associated with crescentic glomerulonephritis in that study [22]. In our study, although the negative predictive value (NPV) of renal dysfunction (defined as serum creatinine > 1.3 mg/dL) was 93% for identifying proliferative LN, the presence of the same does not differentiate between activity and damage. Hence, basing a clinical decision on clinical and biochemical parameters alone will be grossly inadequate, and our study reinforces the importance of renal biopsy at the time of diagnosis.

Various studies have evaluated the predictors of renal response [10,12,23,24,25,26,27]. In our cohort, male sex, higher baseline proteinuria and chronicity scores independently predicted NR at one year. A sensitivity analysis for the missing variables yielded a similar result, ascertaining the validity of our findings. Male sex is an established risk factor for poor renal outcome [28,29]. In a study that assessed the risk factors for the development of chronic kidney disease (CKD) in LN, hypertension at the onset of LN was more common among those patients who developed CKD [30]. Hypertension and high chronicity scores independently predicted NR at one year in an Italian cohort [12]. However, we did not find any association of NR with hypertension, and the long-term effects of the same need further studies. Moreover, immunosuppressive agents are known to reduce blood pressure in SLE [31], and it is crucial to understand whether the persistence of hypertension or refractory hypertension would contribute to CKD progression rather than incident hypertension at diagnosis. Another significant factor in predicting NR at one year in our study was baseline proteinuria. Although the resolution of proteinuria is critical, it is shown that the time taken to complete proteinuria response correlates positively with baseline proteinuria [12]. Our observation is in line with other studies, where the amount of proteinuria at baseline determines the time required for a response [32,33], suggesting that, in individuals with massive proteinuria at baseline, aggressive anti-proteinuria measures in addition to the immunosuppression would be needed. In our study, the use of anti-proteinuria drugs, such as the Renin Angiotensin Aldosterone system (RAS) inhibitors, was not accounted for and may need further exploration.

The importance of histological factors in predicting renal outcomes was assessed by Austin et al. as early as 1984 [34]. The proposed indices for activity and chronicity and at baseline were strongly predictive of the development of renal failure In addition to crescents and fibrinoid necrosis, tubulointerstitial involvement was predictive of poor outcomes [25,35,36,37]. Although our study showed an association of interstitial inflammation and tubular atrophy with NR at one year, when controlled for other factors, such as proteinuria, sex and other histological scores, we observed that a combination of male sex, baseline urine PCR and chronicity score were the main factors determining the one-year outcome. Apart from activity scores and chronicity scores, vascular involvement in the form of thrombotic microangiopathy (TMA) has been associated with adverse outcomes [38]. This finding was not observed in our cohort, probably due to meagre numbers of those with TMA changes.

The strength of our study is the inclusion of a large number of ethnically homogenous Indian patients with biopsy-proven LN from a single centre. The majority of our patients had proliferative LN. Ours is one of the very few studies that looked at a combination of clinical and histological factors in predicting renal outcomes [12,13,24]. The limitations of the study include its retrospective design. The immunosuppressive agents used in Classes I/II and V were dictated based on extrarenal manifestations, as a result of which, their effect cannot be assessed. Although some studies have shown some differences in characteristics with respect to global and segmental subclasses of proliferative LN [39], the same was not assessed in our study. Additionally, the cumulative dose of steroids, other medications used and control of comorbidities, including hypertension, were not factored in this study.

## 5. Conclusions

To conclude, renal biopsy at the onset of LN is important not only in diagnosing LN but also in prognosticating long-term outcomes. Treating patients with suspected LN without histopathological evidence may be a suboptimal approach and may result in the inappropriate treatment of a significant proportion of patients. We propose a combined clinicopathological model including variables such as male sex, baseline UPCR and chronicity score to predict NR at one year. Identifying the baseline predictors of NR would help in the timely escalation of treatment in a treat-to-target approach to improve outcomes. Additionally, it is crucial to augment the treatment regimen with non-immunosuppressive agents to achieve the desired target, especially in those with nephrotic range proteinuria. It would also be important to see how the histological progression of these chronicity and activity indices will determine the renal outcome by using protocolised repeat kidney biopsies.

## Figures and Tables

**Table 1:** Comparison of baseline characteristics among renal biopsy classes in LN patients.

Parameter	Class I/II (n = 52)	Class III/IV (n = 240)	Class V (n = 28)	Combined (III/IV+V) (n = 13)	*p*-Value
**Demography**					
Female, n (%)	46(88.5)	221(92.1)	26(92.9)	14(100)	0.372
Age at SLE onset (median, range)	29(10–57)	25(8–67)	32(11–44)	28(13–47)	0.707
Age at nephritis onset (median, range)	30(11–57)	27(9–67)	33(17–45)	32(13–48)	0.350
Disease duration at enrolment (months) (median, range)	15(0–232)	12(0–228)	24(0–144)	40(0–96)	0.499
**Clinical and biochemical parameters**					
Hypertension, n (%)	8(15.4)	125(52.1)	7(25.0)	8(61.5)	**0.001**
Median Serum Creatinine (mg/dL), Median (IQR)	0.7(0.5–0.86)	0.84(0.68–1.1)	0.65(0.5–0.83)	0.70(0.54–0.79)	**<0.001**
Serum Creatinine > 1.3 g/dL, n (%)	4(7.7)	39(16.3)	2(7.1)	13(100)	**0.044**
eGFR (ml/min/1.73 m2), Median (IQR)	115.8(79.4–152.7)	87.6(62.75–118.8)	130.8(105.7–164.9)	99.5(87.2–126.2)	**<0.001**
eGFR categories, n (%) >90 61–90 30–60 <30	34(65.4) 22(21.2) 5(9.6) 1(1.9)	116(48.3) 67(27.9) 42(17.5) 14(5.8)	23(82.1) 3(10.7) 2(7.1) 0	11(84.6) 2(15.4) 0 0	**0.002**
Serum albumin(g/dL), Median (IQR)	3.2(2.75–3.60)	2.9(2.4–3.3)	2.8(2.48–3.40)	3.0(2.30–3.55)	0.069
Active urinary sediments, n (%)	24(46.2)	154(64.2)	10(35.7)	8(61.5)	**0.006**
UPCR (mg/mg), Median (IQR)	1.2(0.69–1.70)	1.6(0.88–2.79)	1.9(1.5–3.49)	3.2(1.17–6.0)	**<0.001**
UPCR category, n (%) >3 g 2–3 g <2 g	6(11.5) 4(7.7) 41(78.8)	52(21.7) 39(16.3) 144(60.0)	9(32.1) 3(10.7) 15(53.6)	6(46.2) 1(7.7) 6(46.2)	**0.004**
**Disease activity measures**					
Renal SLEDAI, Median (IQR)	4(4–12)	12(8–12)	8(4–8)	8(4–12)	**<0.001**
SLEDAI, Median (IQR)	14(9.5–20.5)	18(14–24)	14(8–18)	14(10.75–16.75)	**0.032**
Extrarenal SLEDAI, Median (IQR)	7 (2–12)	7 (3–13)	4.5 (2–10)	4 (2–10)	0.243
**Serological parameters**					
* Anti-dsDNA high titre positivity, n (%)	23(44.2)	145(60.4)	11(39.3)	7(53.8)	0.849
Low C3/C4, n (%)	34(65.4)	181(75.4)	20(71.4)	10(76.9)	0.838
ACLA/Anti-B2gpI/LAC positivity (any one positive), n (%)	16(30.8)	68(28.3)	7(25.0)	1(7.7)	0.279

Significance at *p*-value < 0.05; LN, Lupus Nephritis; SLE, Systemic Lupus Erythematosus; eGFR, estimated Glomerular Filtration Rate; UPCR, Urine Protein–Creatinine Ratio; SLEDAI, SLE Disease activity Index; dsDNA, double stranded deoxyribonucleic acid; ACLA, anticardiolipin antibody; B2gpI, Beta2 glycoprotein1; LAC, Lupus Anticoagulant; IQR, Inter-Quartile Range. * Titre > 100 U/mL; Low C3 < 0.9 g/L; Low C4 < 0.1 g/L.

**Table 2:** Sensitivity and specificity of baseline parameters in identifying proliferative LN, activity parameters and chronicity.

	Proliferative LN		Crescents		Interstitial Inflammation		Chronicity	
Baseline Parameter	Sensitivity (%)	Specificity (%)	Sensitivity (%)	Specificity (%)	Sensitivity (%)	Specificity (%)	Sensitivity (%)	Specificity (%)
Any one parameter present	96.4	8.7	98.4	4.5	94.5	3.16	95.8	3.85
All four parameters present	7.5	98.7	16.9	95.4	8.8	95.5	11.6	95.6
>1 or <4 parameters present	53.7	81.2	49.2	64.4	41.9	63.9	48.8	67.7

Parameters included: Renal Dysfunction—Serum Creatinine > 1.3 mg/dL, Proteinuria—Spot UPCR > 0.5 mg/mg, Hypertension, Active urinary sediments defined as red blood cells (RBCs) > 5cells/high power field (HPF) or white blood cells (WBC) > 5cells/HPF, or cellular casts that were attributed to SLE,.

**Table 3:** Comparison of baseline characteristics among those who achieved any response (CR/PR) versus non-response (NR) at one year.

Parameter	Responders (CR/PR) (n = 233)	Non-Responders (NR) (n = 60)	*p*-Value
Demography			
Female/male, n (%)	221(94.8)/12(5.2)	48(80)/12(20)	**0.001**
Age at SLE onset, Median (range)	26(10–62)	23.5(12–67)	0.067
Age at nephritis onset, Median (range)	28(11–65)	25(13–67)	0.079
Disease duration at enrolment (months), Median (range)	12(0–232)	18(0–144)	0.770
Duration of follow-up,	37(9–180)	24(2–86)	**0.001**
**Clinical and biochemical parameters**			
Hypertension, n (%)	100(42.9)	34(56.7)	0.061
Creatinine, Median (IQR)	0.79(0.6–0.96)	0.9(0.7–1.33)	**0.009**
Creatinine > 1.3 mg/dL, Median (IQR)	21(9.0)	15(25)	**0.001**
eGFR (ml/min/1.73 m^2^), Median (IQR)	97.2(73.6–136.5)	83.85(50.7–123)	0.203
eGFR categories, n (%) >90 61–90 30–60 <30	137(58.8) 57(24.5) 34(14.6) 4(1.7)	27(45) 15(25) 9(15) 8(13.3)	**0.003**
Serum albumin (mg/dL), Median (IQR)	3(2.5–3.4)	2.9(2.2–3.1)	0.314
Active urinary sediments, n (%)	132(56.7)	44(73.3)	**0.019**
UPCR (g/day), Median (IQR)	1.38(0.8–2.67)	1.95(1.18–4.19)	0.098
UPCR categories, n (%) >2 2–3 <2	51(21.9) 25(11.2) 151(64.8)	18(30) 12(20) 30(50)	0.114
Renal SLEDAI, Median (IQR)	8(4–12)	12(8–12)	**0.014**
Total SLEDAI, Median (IQR)	17(12–22)	16.5(13.7–24.2)	0.852
**Serological parameters**			
* Anti-dsDNA high titre positivity, n (%)	129(55.4)	36(60)	0.658
^$^ Low C3/C4, n (%)	166(71.2)	45(75)	0.347
ACLA/Anti-B2gpI/LAC positivity (any one positive), n (%)	62(26.6)	21(35)	0.401
**Histological**			
Class I/II, n (%)	42(18.0)	3(5)	**0.013**
Class III/IV, n (%)	167(71.7)	49(81.7)	0.117
Class V, n (%)	17(7.3)	5(8.3)	0.788
Combined class, n (%)	7(3.0)	3(5.0)	0.469
Activity score, Median (IQR)	3(1–6)	6(3–9)	**0.001**
Chronicity score, Median (IQR)	0(0–1)	1(0–2)	**0.001**
Chronicity score >3, n (%)	5(2.1)	6(10)	**0.013**
Presence of crescents, no (%)	43(18.5)	17(28.3)	0.104
Fibrinoid necrosis, n (%)	28(12.0)	7(11.7)	0.791
Interstitial inflammation, n (%)	86(36.9)	33(55)	**0.004**
Interstitial fibrosis, n (%)	23(10.7)	9(15)	0.273
Tubular injury, n (%)	44(18.9)	16(26.7)	0.130
Tubular atrophy, n (%)	64(27.5)	27(45)	**0.003**
Blood vessel changes, n (%) Fibrinoid necrosis Other changes **	2(0.9) 206(88.4)	1(1.7) 50(83.3)	0.606 0.339

Significance at *p*-value < 0.05; ** Intimal fibrosis, Medial hypertrophy, Arteriolar hyalinosis, arteriosclerosis; LN, Lupus Nephritis; SLE, Systemic Lupus Erythematosus; eGFR, estimated Glomerular Filtration Rate; UPCR, Urine Protein–Creatinine Ratio; SLEDAI, SLE Disease activity Index; dsDNA, double stranded deoxyribonucleic acid; ACLA, anticardiolipin antibody; B2gpI, Beta2 glycoprotein1; LAC, Lupus Anticoagulant; IQR, Inter-Quartile Range. * Titre > 100 U/mL; ^$^ Low C3 < 0.9 g/L; Low C4 < 0.1 g/L.

**Table 4:** Univariate and multivariate logistic regression analyses: Baseline predictors of non-response to therapy at one year.

	Univariate Analysis			Multivariate Analysis		
	OR	95% CI	*p*-Value	OR	95% CI	*p*-Value
Male	4.6	1.9–10.8	**<0.001**	3.9	1.4–11.0	**0.008**
Age at SLE onset, years	0.97	0.9–1.0	0.080	0.9	0.9–1.0	0.053
Hypertension	0.5	0.3–1.0	0.063	1.1	0.5–2.3	0.721
Serum Creatinine, mg/dL	2.7	1.6–4.6	**<0.001**	1.9	1.1–3.2	**0.041**
Serum albumin, mg/dL	0.6	0.4–0.9	**0.048**			
Active urinary sediments	0.4	0.2–0.8	**0.020**	0.7	0.3–1.6	0.479
UPCR g/day	1.1	1.0–1.3	**0.002**	1.2	1.0–1.3	**0.002**
Anti-dsDNA high positivity *	1.1	0.5–2.3	0.658			
Low C3/C4 ^$^	0.7	0.3–1.4	0.349			
ACLA/Anti-B2gpI/LAC positivity	1.3	0.7–2.4	0.402			
Activity score	1.1	1.0–1.2	**<0.001**	1.0	0.7–1.1	0.145
Chronicity score	1.6	1.2–2.0	**<0.001**	1.5	1.2–2.0	0.001
Crescents	0.5	0.3–1.1	0.107			
Fibrinoid necrosis	1.0	0.4–2.5	0.906			
Interstitial inflammation	0.3	0.2–0.7	**0.004**			

Significance at *p*-value < 0.05; * Titre > 100 U/mL; ^$^ Low C3 < 0.9 g/L; Low C4 < 0.1 g/L; OR, Odds Ratio; CI, confidence interval; SLE, Systemic Lupus Erythematosus; UPCR, Urine Protein–Creatinine Ratio; dsDNA, double stranded Deoxyribonucleic Acid; ACLA, anticardiolipin antibody; B2gpI, Beta2 glycoprotein1; LAC, Lupus Anticoagulant.

## Data Availability

All the data are available from the corresponding author and will be made available upon reasonable request.

## Supplementary Materials

The following supporting information can be downloaded at: https://www.mdpi.com/article/10.3390/diagnostics12123163/s1.
