# Supplementary material for: Performance of Clinical and Biochemical Parameters in Identifying Renal Histopathology and Predictors of One-Year Renal Outcome in Lupus Nephritis—A Single Centre Study from India
Source: Diagnostics (Basel). 2022 Dec 14;12(12):3163. doi: 10.3390/diagnostics12123163 (PMC9777017; doi:10.3390/diagnostics12123163)
Supplement: Supplementary file 1 [file diagnostics-12-03163-s001.zip › diagnostics-1962128-supplementary.pdf]

Supplementary Table S1: Sensitivity and specificity of baseline parameters to identify proliferative LN, activity parameters and chronicity

	Proliferative LN				Crescents				Interstitial inflammation				Chronicity			
**Baseline parameter**	**Sensitivity (%)**	**Specificity (%)**	**PPV (%)**	**NPV (%)**	**Sensitivity (%)**	**Specificity (%)**	**PPV (%)**	**NPV (%)**	**Sensitivity (%)**	**Specificity (%)**	**PPV (%)**	**NPV (%)**	**Sensitivity (%)**	**Specificity (%)**	**PPV (%)**	**NPV (%)**
Hypertension	53.4	78.2	0.04	99.9	64.4	58.0	0.02	99.9	53.9	58.4	0.02	99.9	60.5	61.8	0.02	99.9
Renal dysfunction	15.4	92.5	0.03	99.9	21.5	88.5	0.03	99.9	18.3	91.7	0.03	99.9	21.1	91.3	0.04	99.9
Proteinuria	88.5	77.1	0.06	99.9	90.6	13.2	0.02	99.9	88.8	12.4	0.02	99.9	90.6	14.5	0.02	99.9
AUS	82.6	66.4	0.02	99.9	86.6	48.2	0.02	100.0	58.0	41.1	0.01	99.9	60.0	42.3	0.02	99.9
HTN, proteinuria	45.5	83.7	0.04	99.9	61.1	65.0	0.03	99.9	50.0	66.6	0.02	99.9	55.5	68.3	0.03	99.9
HTN, AUS	34.3	87.5	0.04	99.9	55.3	75.8	0.03	99.9	25.1	73.5	0.02	99.9	39.5	74.4	0.02	99.9
HTN, Renal dysfunction	11.4	96.3	0.05	99.9	21.5	92.7	0.04	99.9	14.0	94.7	0.04	99.9	16.8	93.8	0.04	99.9
Renal dysfunction, proteinuria	11.1	90.3	0.05	99.9	20.0	93.1	0.04	99.9	15.4	96.2	0.06	99.9	55.5	51.1	0.02	99.9
Renal dysfunction, AUS	11.5	95.0	0.03	99.9	16.9	92.0	0.03	99.9	12.5	93.0	0.03	99.9	15.8	93.7	0.04	99.9
Proteinuria, AUS	53.7	71.2	0.02	99.9	79.6	56.4	0.03	99.9	54.4	51.0	0.02	99.9	55.7	51.5	0.02	99.9
Renal dysfunction, AUS, HTN	53.4	78.2	0.04	99.9	16.9	95.2	0.05	99.9	53.9	58.4	0.02	99.9	11.6	95.8	0.04	99.9

AUS, Active Urinary Sediments; HTN, Hypertension, LN, Lupus Nephritis; NPV, Negative Predictive Value, PPV, Positive Predictive Value

Supplementary Table S2: Comparison of baseline characteristics among those with and without crescents, fibrinoid necrosis, interstitial inflammation in proliferative LN

Parameter	Crescents			Fibrinoid necrosis			Interstitial inflammation		
	Present (n = 62)	Absent (n = 174)	P value	Present (n = 36)	Absent (n = 201)	P value	Present (n = 112)	Absent (n =99)	P value
Female, n (%)	57(91.9)	160(92)	0.996	32(88.9)	186(92.5)	0.689	102(91.1)	92(92.9)	0.621
Hypertension, n (%)	40(64.5)	84(48.3)	**0.041**	24(66.7)	100(49.8)	**0.034**	60(53.6)	50(50.5)	0.666
Serum Creatinine (mg/dL), Median (IQR)	0.9(0.7-1.3)	0.8(0.6-1.1)	0.278	0.8(0.6-1.1)	0.8(0.7-1.1)	0.526	0.9(0.7-1.2)	0.7(0.6-1.0)	**<0.001**
Serum Creatinine >1.3g/dL, n (%)	14(22.4)	24(13.8)	0.106	5(13.9)	24(16.9)	0.773	23(20.5)	9(9.1)	**0.021**
eGFR, Median (IQR)	80.2(54.3-114.6)	91.5(67-125)	0.254	84.8(63.5-133.3)	86.4(62-115.8)	0.806	79.2(56.4-104.7)	98(69.4-142.9)	**<0.001**
Active urinary sediments, n (%)	54(87.1)	97(55.7)	**<0.001**	25(69.4)	127(63.2)	0.277	63(56.3)	71(71.7)	**0.020**
UPCR (mg/mg), Median (IQR)	2.3(1.2-4.3)	1.4(0.8-2.3)	**<0.001**	2.2(1.1-5.1)	1.5(0.8-2.5)	**0.010**	1.8(1.0-3.4)	1.2(0.7-2.5)	**0.023**
Renal SLEDAI, Median (IQR)	12(8-12)	8(8-12)	**<0.001**	12(8-12)	12(8-12)	0.213	8(8-12)	12(8-12)	**0.003**
SLEDAI, Median (IQR)	18(14-22)	18(13-24)	0.886	19(14-23)	18(14-24)	0.911	18(13-24)	19(14-24)	0.292
*Anti- dsDNA high titer positivity, n (%)	36(58.1)	108(62.1)	0.359	20(55.6)	125(62.2)	0.879	63(56.3)	63(63.6)	0.133
Low C3/C4, n (%)	48(77.4)	130(74.7)	0.845	29(80.6)	150(74.6)	0.053	80(71.4)	7(77.8)	0.708
ACLA/Anti-B2gpI/LAC positivity (any 1 positive), n (%)	20(32.3)	47(27.0)	0.502	10(27.8)	57(28.4)	0.800	29(25.9)	33(33.3)	0.274

*Titre > 100 U/mL; $-Low C3 <0.9g/L; Low C4 <0.1 g/L; IQR, Inter-Quartile Range; SLEDAI Systemic Lupus Erythematosus Disease Activity Index; UPCR, Urine Protein-Creatinine Ratio; dsDNA, double stranded DeoxyriboNucleic Acid; ACLA, anticardiolipin antibody, B2gpI, Beta2 glycoprotein1; LAC, Lupus Anticoagulant;

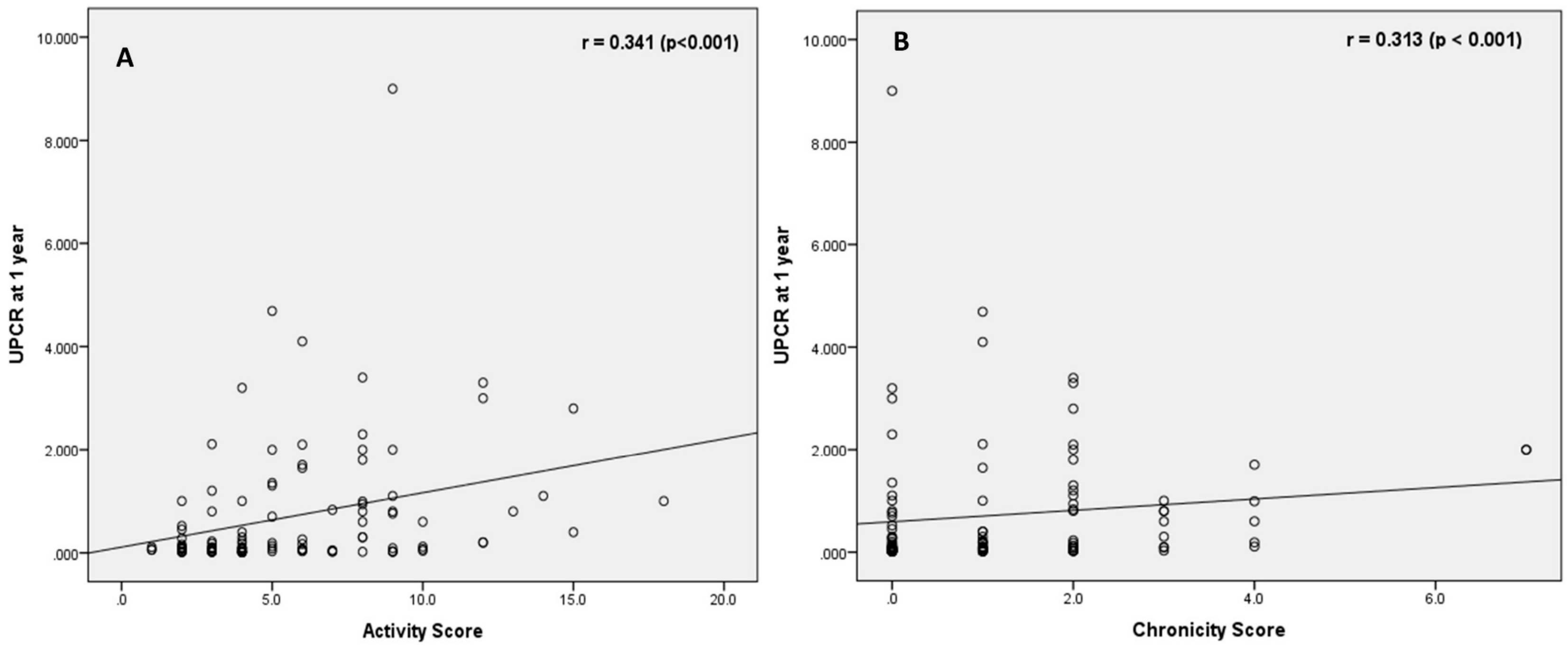

Figure S1: Correlation between A) Activity score and UPCR at 1 year; B) Chronicity score and UPCR at 1 year
